# Validation of an In Vitro Diagnostic Test for Endometriosis: Impact of Confounding Medical Conditions and Lesion Location

**DOI:** 10.3390/ijms25147667

**Published:** 2024-07-12

**Authors:** Elza Daoud, David F. Archer, Fabio Parazzini, Bárbara Herranz-Blanco

**Affiliations:** 1 Chemo Research, 28050 Madrid, Spain; elza.daoud@exeltis.com; 2 Department of Obstetrics and Gynecology, Eastern Virginia Medical School, Norfolk, VA 23407, USA; archerdf@evms.edu; 3 Department of Clinical Science and Community Medicine, University of Milan, 20122 Milan, Italy; fabio.parazzini@unimi.it

**Keywords:** in vitro diagnostic test, endometriosis, validation, lesion location, superficial endometriosis, confounding conditions

## Abstract

With the aim to shorten the time for diagnosis and accelerate access to correct management, a non-invasive diagnostic test for endometriosis was developed and validated. The IVD test combines an ELISA test kit to quantify CA125 and BDNF concentrations in serum and a data treatment algorithm hosted in medical software processing results from the ELISA test and responses to six clinical variables. Serum samples and clinical variables extracted from psychometric questionnaires from 77 patients were collected from the Oxford Endometriosis CaRe Centre biobank (UK). Case/control classification was performed based on laparoscopy and histological verification of the excised lesions. Biomarkers serum concentrations and clinical variables were introduced to the software, which generates the qualitative diagnostic result (“positive” or “negative”). This test allowed the detection of 32% of cases with superficial endometriosis, which is an added value given the limited efficacy of existing imaging techniques. Even in the presence of various confounding medical conditions, the test maintained a specificity of 100%, supporting its suitability for use in patients with underlying medical conditions.

## 1. Introduction

Endometriosis is a progressive, estrogen-dependent disease that affects approximately 10% of women of reproductive age [1]. It is characterized by the presence of endometrial-like tissue outside the uterus, commonly affecting the pelvic cavity, ovaries, fallopian tubes, and other surrounding structures [2]. These lesions result in a chronic inflammatory response, which can lead to the formation of scar tissue and adhesions [3]. The clinical presentation of endometriosis can be very diverse, with a wide range of symptoms, including chronic non-menstrual pelvic pain, dysmenorrhea, dysuria, infertility, and many others, with the onset of symptoms usually occurring during adolescence [1,4]. The severity and manifestation of symptoms can be influenced by various factors, including the location and extent of the endometrial implants, hormonal fluctuations, and individual pain thresholds [5,6]. Also, symptoms often overlap with those of various other conditions [7,8]. In clinical practice, diagnostic laparoscopy is no longer considered necessary to start the treatment of women with suspected endometriosis. Empirical treatment, including first-line hormonal contraceptives or progestogens, can be considered in women with suspected endometriosis who do not wish to conceive. Instead, laparoscopy should be considered for the diagnosis and treatment of endometriosis for patients with negative imaging results or where empirical treatment was unsuccessful [9].

Therefore, clinicians should rely on clinical examination in conjunction with imaging techniques such as transvaginal ultrasound (TVUS) and magnetic resonance imaging (MRI) for differential diagnosis. In this scenario, the delay in the appropriate medical management of the patients becomes relevant, including empirical treatment and counselling. Potential causes for this delay have been pinpointed and include quality and availability of imaging equipment along with the continued lack of awareness of the disease among the general population and medical community [9]. TVUS and MRI have been shown to accurately diagnose some endometriosis cases, but these are usually limited to more severe stages of the disease [10,11]. Still, despite the recommendations for clinical practice, diagnostic laparoscopy with histological confirmation remains the most accurate method for diagnosing the disease [1,2,3,12]. The delay in the identification of early stages allows the condition to progress, leading to increased severity, fibrosis, and potential infertility [13,14]. In this context, developing a non-invasive diagnostic test for endometriosis becomes crucial [15,16].

Prior studies have delved into an extensive array of biomarkers, highlighting the complexity of understanding endometriosis. Cancer antigen 125 (CA125), a widely recognized glycoprotein, has been a focal point in research due to its association with various gynecological conditions, including endometriosis [17,18,19]. CA125 is known to be expressed on the cell surface of some derivatives of embryonic coelomic epithelium, which are believed to be the precursors of endometriotic lesions [20]. Despite its usefulness, the lack of specificity and limited sensitivity as a standalone marker, along with its utility being limited to late stages of the disease, has underscored the need for complementary biomarkers. Brain-derived neurotrophic factor (BDNF), known for its involvement in neuroplasticity and neuronal survival, has emerged as a promising candidate, with studies demonstrating elevated levels in patients with endometriosis compared to healthy controls [11,21,22]. BDNF is a neurotrophin that plays a role in the uterine physiology [21] by binding to receptor NTRK2, also known as TrKB. The expression of BDNF and NTRK2 has been found to be significantly higher in the uterus of women with endometriosis than in healthy controls [12]. Moreover, BDNF was found to be overexpressed in the ectopic but not in the eutopic endometrial tissue. Estrogens induce BDNF production by macrophages, and BDNF promotes neurogenesis by binding to NTRK2 receptors on nerves. Estrogens also induce the release of pro-inflammatory mediators from mast cells, which sensitize the nerve endings in the endometriotic lesions and cause pain [23,24]. However, the lack of specificity among individual biomarkers emphasizes the necessity of a comprehensive diagnostic approach integrating multiple markers to enhance accuracy and reliability in endometriosis detection.

Recently, we have developed a diagnostic treatment algorithm that combines CA125 and BDNF measurements with six pertinent clinical variables: patient’s surgical history related to endometriosis, the manifestation of painful periods as a leading symptom for endometriosis referral, the intensity of menstrual pain during the previous cycle, the age at the onset of intercourse-related pain, the age at the initiation of regular painkiller usage, and the age at the initial diagnosis of an ovarian cyst. CA125, BDNF, and the six clinical factors were integrated into the final logistic regression model, achieving an AUC of 0.867, sensitivity of 51.5%, and specificity of 95.6% [25]. The mean BDNF serum concentrations in endometriosis and healthy patients were 21.66 ng/mL (SD 6.02) and 19.06 ng/mL (SD 4.81), respectively. The mean CA125 serum concentrations in endometriosis and healthy patients were 41.3 IU/mL (SD 38.8) and 15.8 IU/mL (SD 15.7), respectively.

The influence of confounding conditions on the final diagnosis of endometriosis using this test was challenged. This is because multiple conditions, gynecological (for instance, adenomyosis [26,27,28,29], pelvic inflammatory disease (PID) [30,31,32], uterine fibroids [32,33,34], and ovarian cysts [32,35]) and non-gynecological (for instance, inflammatory bowel disease (IBD) [36], rheumatoid arthritis [37,38,39], asthma [40], anxiety and depression [41,42,43]) could affect the levels of CA125 and BDNF.

The primary aim of this study was to validate the diagnostic performance of the test in endometriosis patients while also discerning the specific subgroup of patients in which the test demonstrates superior performance. The secondary aim was to further investigate how confounding conditions influence CA125 and BDNF and whether the performance of the test is affected.

## 2. Results

### 2.1. Diagnostic Performance by Endometriosis Lesion Type

One hundred percent of controls from the validation dataset were correctly diagnosed (negative) with the IVD test, based on the threshold established in the development dataset. With this, a sensitivity (after weighing for disease stages) of 46.2% (95% CI: 25.5–66.8%) and a specificity of 100% (95% CI: 86.7–100%) was obtained. The accuracy was 64.1% (95% CI: 50.4–77.8%) and the AUC was 0.758 (95% CI: 0.650–0.867). To understand in which subgroup of endometriosis patients the test works best, i.e., is capable of detecting the highest number of cases, patients were separated into subgroups by lesion types. First, the association between the stages of endometriosis and the types of endometriosis lesions was examined using Pearson’s chi-squared test. The analysis revealed a significant association (χ^2^ = 765.76, df = 25, *p* < 0.001), indicating a strong relationship between the revised American Society for Reproductive Medicine (rASRM) stages classification of endometriosis and classification by types of lesions. The contingency table (Table 1) provides insight into how lesion types are distributed by endometriosis rASRM stage for patients of pooled development and validation datasets. Superficial lesions are observed mostly in stage I (81.5%). Extended lesions (endometrioma + DIE) are mostly observed in stage IV.

Sensitivity was investigated by lesion type. Results (as reported in Table 2) indicate that the IVD test successfully identified around half of the cases of DIE and endometrioma. Furthermore, with a sensitivity of 69.70%, the IVD test demonstrates that the test works best in identifying cases of DIE + endometrioma. Interestingly, 32% of cases of superficial endometriosis were correctly identified with the test. As expected, the two cases of endometriosis located within c-section scars could not be identified with the test (different pathophysiology, as described above).

**Figure 1:**
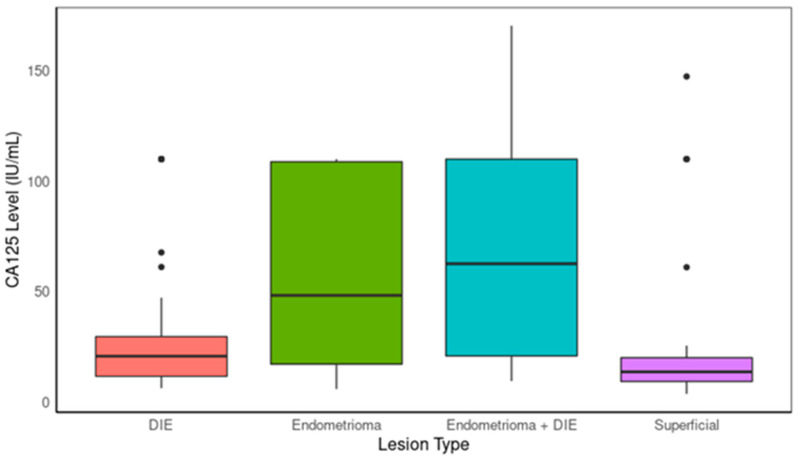
Boxplot comparing CA125 levels between lesion types.

An ANOVA was conducted to examine the differences in CA125 values among various types of endometriosis lesions in the pooled datasets (development and validation datasets, Figure 1). The results revealed a significant effect of lesion type on CA125 levels (F(5, 275) = 26.162, *p* < 0.001). Post hoc analyses indicated that the differences were statistically significant (*p* < 0.001) across the various lesion types. The Tukey multiple comparison of means at a 95% family-wise confidence level revealed several significant differences between the types of lesions in terms of CA125 levels: comparing endometrioma to DIE, there was a statistically significant difference (*p* < 0.01). Additionally, the mean CA125 level (56.05 IU/mL, SD = 39.35) was higher for endometrioma than for DIE (32.28 IU/mL, SD = 32.69) (*p* = 0.01). Moreover, the mean CA125 level for endometrioma + DIE (67.69 IU/mL, SD = 45.49) was higher than the mean CA125 level for DIE (mean = 32.28 IU/mL, SD = 32.69) (*p* < 0.001). Lower CA125 levels were observed for superficial lesions (mean = 19.55 IU/mL, SD = 24.74) than for endometrioma (*p* < 0.001), DIE (*p* = 0.02), and endometrioma + DIE (*p* < 0.001). An ANOVA conducted on BDNF values across different lesion types did not show any significant differences of BDNF across different types of lesions (*p* = 0.094). This suggests that the improved sensitivity for DIE + endometrioma lesions is likely to be due to higher levels of CA125 in those lesions, contributing to a higher rate of true positive results in cases with those lesions.

### 2.2. Interference of Potentially Confounding Medical Conditions

As shown in Table 3, despite 76% (19 out of 25 controls) of controls in the validation dataset having at least one condition that could elevate CA125, the specificity of the diagnostic test was 100%.

Two-way ANOVA with EndoState (Cases/controls) and each confounding condition as predictors was run on CA125 levels in the pooled datasets. For ovarian cysts, the ANOVA revealed the main effect of EndoState (F = 32.97, *p* < 0.001) and ovarian cyst condition (F = 22.65, *p* < 0.001) on CA125 levels. Individuals with ovarian cysts had higher CA125 values than individuals without ovarian cysts (*p* < 0.001). No interaction between both predictors was reported. For uterine fibroids (UF), a main effect for condition on CA125 was observed (F = 11.22, *p* < 0.001) as well as an expected main effect for EndoState (F = 15.30, *p* < 0.001). No interaction between both predictors was reported. Individuals with uterine fibroids had higher CA125 values than individuals without uterine fibroids (*p* < 0.001).

Two-way analysis of variance (ANOVA) with EndoState (Cases/controls) and each confounding condition as predictors was run on BDNF in pooled datasets. For chronic fatigue only, a main effect was observed for EndoState (F = 5.75, *p* = 0.017) and an interaction between EndoState and the condition (F = 4.20, *p* = 0.04). Pairwise comparisons revealed that controls with chronic fatigue had lower BDNF values than cases without chronic fatigue (mean difference = 6.06, *p* = 0.04). A Kruskal–Wallis test to assess the difference in BDNF between the different BMI groups revealed statistically significant differences (*p* = 0.01). A Mann–Whitney U test for pairwise comparisons revealed that BDNF was significantly elevated in obese individuals (>30 kg/m^2^), compared to overweight (25–30 kg/m^2^; *p* = 0.01) and upper-normal weight individuals (22.5–25 kg/m^2^; *p* = 0.001).

The performance of the diagnostic test was determined in the validation dataset excluding each confounding condition at a time. Results, as shown in Table 4, indicate that the sensitivity values when excluding conditions stay within the 95% CI of the original sensitivity (all conditions included) between 34.3 and 62.9, meaning that no condition critically affects the performance of the test.

## 3. Discussion

The newly developed test for endometriosis demonstrates a high specificity of 100%, suggesting its potential use as a rule-in test in clinical practice. This diagnostic test could significantly contribute to the initial diagnostic workup, effectively confirming the presence of endometriosis and providing clinicians with a reliable tool for early detection and intervention. Moreover, the test demonstrates an encouraging ability to identify superficial lesions of endometriosis, as evidenced by the reported sensitivity of 32%. This feature is of particular significance considering the constraints associated with the ability of alternative diagnostic methods to detect superficial lesions: superficial endometriosis, characterized by its subtle and less invasive nature, presents unique challenges for detection using ultrasound or MRI. Peritoneal implants invading less than 5 mm of depth from the peritoneal surface are often invisible on MRI [11]. These imaging techniques may struggle to capture the nuanced characteristics of these lesions due to their limited ability to visualize subtle changes in the peritoneum and pelvic surfaces [44]. Additionally, the lack of specific imaging markers or distinguishing features that differentiate superficial lesions from surrounding healthy tissue makes it difficult to accurately identify these lesions using standard imaging modalities. The intricate anatomical location of superficial lesions, often nestled within complex pelvic structures, further contributes to the complexity of their detection, as these areas may be challenging to access and visualize accurately using traditional imaging approaches [45]. By enabling the identification of superficial lesions, the test offers clinicians an essential means of identifying cases that would otherwise have gone undetected, thereby facilitating a more comprehensive and accurate patient management.

Also, the test demonstrated a relatively high sensitivity of 69.70% in detecting endometrioma+ DIE lesions, possibly correlated to patients with those lesions having the highest level of CA125 compared to other types of lesions. Endometrioma, an endometriosis-related ovarian cyst, often exhibits elevated CA125 levels due to its involvement of the ovaries and resulting inflammatory processes. The higher mean CA125 level observed in this group aligns with prior studies [46]. The observed higher mean CA125 level in the endometrioma + DIE lesions compared to the DIE alone, along with the lowest CA125 levels in the superficial endometriosis, suggests that CA125 expression increases with the extent of the disease (i.e., the extent of tissue involvement and disease spread).

The test input consists of various parameters contributing to the diagnosis, which have been discussed in detail in a previous publication [25]. This may lead to the fact that even in the presence of confounding medical conditions that altered the levels of either CA125 or BDNF, the overall test performance is preserved.

Some limitations of the current study exist. The medical conditions examined are limited to those observed in the population included in the studies. Further, analysis was only carried out when the prevalence of the condition was above 1%. Therefore, it is not possible to draw significant conclusions regarding the impact of conditions with a prevalence < 1% on the overall test performance. Another limitation of the test is that it has been developed and further validated in blood samples obtained prior to the excision surgery, and there is currently not available information to assess the outcome of the surgical procedure.

In summary, even in the presence of various confounding medical conditions, the test maintains its robustness and reliability, emphasizing its independence from potential confounding factors with 100% of the controls being negative. This characteristic supports its suitability for use in various clinical settings, irrespective of the patient’s medical history, thereby ensuring its applicability without contraindications.

## 4. Materials and Methods

### 4.1. Patients’ Characteristics and Classification

The current report is a prospective analysis study using biobank samples. A total of 281 samples extracted from the renowned Oxford Endometriosis CaRe Centre biobank in the UK were included for the development (I) and external validation (II) studies. The biobank’s repository comprised meticulously curated serum samples and comprehensive clinical information derived from pre-surgical assessments and post-operative procedures of patients within reproductive age (18–50 years old) undergoing laparoscopy because of suspected endometriosis. Patients were classified as cases or controls based on laparoscopy and thorough evaluation of histological findings. After undergoing laparoscopy, patients diagnosed with endometriosis were categorized into stages according to the rASRM classification. Patients who had not used hormones in the 3 months prior to surgery were selected.

A total of 136 endometriosis cases and 68 controls were included in the development study (n = 204). For the validation study (n = 77), 52 cases and 25 controls were included. The demographic characteristics of those patients are available in Table 5. The experimental procedures received approval from the Ethics Committee of CEIm HM Hospitales (codes: 19.05.1411-GHM and 22.03.2001-GHM).

### 4.2. Lesion Location and Subtyping

Imaging findings and surgical examinations have been reported for each subject included in the study. Endometriosis lesions were investigated by location. From these findings, endometriosis lesions were classified into subgroups according to their location in the ovaries and the peritoneal cavity: superficial (<5 mm depth), endometrioma, and/or deep infiltrative endometriosis (DIE). Specifically, the designation “superficial” was assigned when only superficial endometriosis lesions were identified in the ovaries or peritoneal cavity. The classification of “endometrioma” was used when endometriomas were detected in the ovaries, either with or without accompanying superficial endometriosis. In cases where infiltrative lesions were observed in the peritoneal cavity, with or without associated superficial endometriosis, lesions were classified as “DIE”. Moreover, the “endometrioma + DIE” classification was assigned when both DIE and endometriomas were found in the peritoneal cavity, with or without superficial endometriosis. While endometriosis is thought to be caused by retrograde menstruation, the most likely cause of caesarean section (c-section) scar endometriosis is iatrogenic implantation. Due to this different aetiology, 2 patients with c-section scar endometriosis were misclassified as they should fall under a different category than endometriosis with spontaneous implantation. The distributions of cases of the development and validation studies by lesions type are described in Table 6.

### 4.3. Confounding Disease Screening

Patients were asked to fill out a presurgical survey including a question to indicate the absence/presence of confounding medical conditions from a list. They were asked to please mark whether they have had any of the following medical conditions, and at what age they were first diagnosed by a doctor (please tick all that apply)”, and were given the list of medical conditions. Patients were also asked to indicate whether they were affected by other unlisted medical conditions. This survey was administered to patients in one of its 3 versions: version #1 did not list 3 medical conditions: anxiety (1), cardiovascular disease (2) and high blood pressure (3). These conditions were only listed in questionnaires #2 and #3. Versions #2 and #3 were responded by 141 out of 190 patients included in the development study (14 patients did not answer to this question out of 204) and 64 out of 77 patients included in the validation study. For completeness, imaging and surgical findings were used to further identify patients with gynaecological conditions. With respect to BMI, the information was available for 201 out of 204 patients included in the development study and 75 out of 77 from the validation study.

Table 7 depicts the prevalence of the confounding conditions in patients included in the development and validation studies.

### 4.4. Blood Sample Collection and Biomarkers Measurement

The specimens were gathered and managed with explicit patient consent, following the guidelines outlined in the Standard Operating procedures of the World Endometriosis Research Foundation [47]. Before the collection of blood, patients were instructed to maintain a minimum fasting period of 10 h. The serum samples were then preserved in the biobank at temperatures as low as −80 °C for a duration of up to 5 years, after which they were transferred to the designated laboratory for analysis. The ELISA utilized in this in vitro diagnostic test functions as a solid-phase sandwich enzyme immunoassay for the precise determination of BDNF and CA125 levels within human serum [25].

### 4.5. Data Treatment Algorithm

All the necessary input parameters, including serum CA125, serum BDNF, and clinical variables, were gathered. Subsequently, laboratory technicians input this data into the IVD test diagnostic medical software, which houses the data treatment algorithm. The algorithm processed the input and generated outcomes, classifying them as either positive or negative based on whether the value exceeded or fell below the predetermined threshold value, respectively.

### 4.6. Statistical Analysis

Statistical analysis was conducted utilizing R software, version 4.1.3, provided by the R Foundation for Statistical Computing in Vienna, Austria. The statistical significance level was set at *p* < 0.05, indicating a threshold below which results were considered statistically significant. In the validation study, the IVD test software Version 1 (was utilized to compute algorithm scores and their corresponding outcomes. These outcomes were delineated as positive diagnosis when the score surpassed the defined cut-off, and negative diagnosis when the score fell below the defined cut-off. Specifically, the validation study’s sensitivity and specificity were expected to align with or exceed the lower limits of the sensitivity and specificity 95% confidence intervals outlined in the algorithm development study: an AUC of 0.867 with a sensitivity of 51.5% (42.8–60.1) at a specificity of 95.6% (86.8–98.9%) as reported by Herranz et al. To assess the IVD test clinical performance, the results of the primary performance parameters (sensitivity and specificity) were contrasted with the acceptance criteria values established during the development study. To ensure equitable representation of both the low-stage and high-stage groups, the outcomes in the validation were appropriately weighted.

To further elucidate the performance of the IVD test, the sensitivity for each endometriosis classification, with the values specified alongside their respective 95% CI, is reported for the distinct subgroups based on lesion types. For a more comprehensive assessment of the test’s efficacy over a larger sample size, development and validation datasets were pooled. Analysis was run on a pooled dataset. BDNF values in pooled datasets followed a normal distribution and CA125 values were arithmetically transformed to follow a normal distribution. To investigate the effect of confounding diseases on biomarkers levels and the performance of the test, only conditions with >1% prevalence in both datasets were considered. A two-way ANOVA analysis was conducted to assess the effect of medical conditions and EndoState (Cases/controls) on CA125 and BDNF, respectively, including an interaction term. In addition, in accordance with the World Health Organization classification [48] that considers underweight, overweight, and obesity as medical conditions, we investigated the association of BDNF and CA125 with BMI. For this, BMI was categorized into four groups: <22.5 (underweight and lower-normal), >22.5 ≤25 (upper-normal), >25 ≤30 (overweight), and >30 (obese) kg/m^2^. As BMI is not normally distributed, non-parametric tests were applied. A Kruskal–Wallis test was used to assess the difference between the different groups. A Mann-Whitney U test was applied for pairwise comparisons between categories. Only results for the categories underweight/lower-normal weight, overweight, and obese are presented as medical conditions. Only conditions showing significant main effects or interaction will be reported. Furthermore, the performance of the algorithm on validation data was evaluated after excluding each specific condition, one at a time.

## 5. Conclusions

Overall, the high specificity of the test, coupled with its independence from potential confounding medical conditions, positions it as a valuable and reliable tool contributing to the accurate and timely management of endometriosis.

## Figures and Tables

**Table 1:** Contingency table for the distribution of lesion types by endometriosis rASRM stages.

rASRM Stage Lesion Type	Stage I (n = 81)	Stage II (n = 30)	Stage III (n = 34)	Stage IV (n = 41)	Unclassified (n = 2)
C-section	0	0	0	0	2
DIE	7	17	7	9	0
Endometrioma	5	0	16	7	0
Endometrioma + DIE	0	3	10	20	0
Superficial	66	10	1	2	0
Unclassified	3	0	0	3	0

**Table 2:** Distribution of cases, number of true positive, and sensitivity by lesion type in both development and validation datasets.

Endometriosis Classification	Number of Cases (Development and Validation) (*n* = 188)	Number of Correct Diagnosis (True Positives)	Sensitivity (95% CI)
Superficial	78	25/78	32.05 (21.93–43.58)
Endometrioma	28	13/28	46.43 (27.51–66.13)
DIE	41	21/41	51.22 (35.13–67.12)
DIE + endometrioma	33	23	69.70 (51.2–84.4)
C-section	2	0	0
Unclassified	6	1	16.67 (0.42–64.12)

**Table 3:** Distribution of gynecological conditions known to elevate CA125 across controls.

Gynecological Condition	Number of Controls in Development Data (n = 68)	Number of Controls in Validation Data (n = 25)	Total Number of Controls (n = 93)
Ovarian cysts	28	11	39
Uterine fibroids	7	3	10
Adenomyosis	0	1	1
PCOS	16	8	24
Pelvic inflammatory disease	4	2	6
At least one condition	40	19	59

**Table 4:** Performance of the IVD test (validation dataset) excluding each medical condition at a time.

Left Out Condition	Number of Subjects by Condition	Sensitivity	95% CI Lower Limit	95% CI Upper Limit
All (no data left out)	0	48.5	34.3	62.9
Ovarian cyst	27	50.8	34.1	67.4
Uterine fibroids	7	45.8	31.3	61
Adenomyosis	2	46.1	32	60.8
Depression requiring medication or therapy	29	34.3	18.9	53.4
Anxiety requiring medication or therapy	20	37.9	22.6	55.8
Pelvic Inflammatory Disease	6	49.8	35.8	63.9
Eczema	16	55.6	39.6	70.5
Polycystic Ovary Syndrome	17	48.6	33.1	64.3
Interstitial cystitis	7	48.9	33.9	64
Asthma	23	48.8	32.1	65.7
Chronic fatigue syndrome—Myalgic encephalomyelitis	1	47.6	33.4	62.2
Fibromyalgia	1	47.6	33.4	62.2
Irritable Bowel Syndrome	17	42.7	27.2	59.6
Migraine	22	42	26	59.6
Glandular fever	5	47.9	33.5	62.6
Ulcerative colitis	2	46.1	32	60.8
High blood pressure	4	47.9	33.5	62.6
BMI ≤ 22.5 (underweight/lower-normal)	16	47.1	30.5	64.4
BMI > 25 ≤ 30 (overweight)	16	53.8	36.2	70.6
BMI > 30 (obese)	8	46.8	31.8	62.5

**Table 5:** Demographic characteristics and rASRM classification of the patients in the development (I) and validation (II) studies.

	Development Study (I)		Validation Study (II)	
	Controls n = 68	Cases n = 136	Controls n = 25	Cases n = 52
Age years (mean ± SD)	33.5 (5.96)	35.6 (6.42)	35 (6.44)	35 (6.47)
BMI (mean ± SD)	25.38 (4.63)	26.46 (5.32)	26 (5.23)	26 (5.14)
rASRM classification				
I–II		68 (50%)	-	42 (81%)
III–IV	-	68 (50%)	-	7 (13%)
Missing information	-	-	-	3 (6%)

**Table 6:** Classification of endometriosis cases according to lesion location.

Endometriosis Classification	Development Study (I) n = 136	Validation Study (II) n = 52
Superficial	54 (39.7%)	24 (46.2%)
Endometrioma	25 (18.4%)	3 (5.8%)
DIE	28 (20.6%)	13 (25%)
DIE + endometrioma	25 (18.4%)	8 (15.4%)
Unclassified	4 (2.9%)	2(3.8%)
C-section scar	0	2 (3.8%)

**Table 7:** Prevalence of confounding conditions in the development and validation datasets.

Confounding Condition	Prevalence in Development Study	Prevalence in Validation Study
Anxiety requiring medication or therapy	39/141 (28%)	20/64 (31%)
Asthma	42/190 (22%)	23/77 (30%)
Adenomyosis	7/190 (3.7%)	2/77 (2.6%)
Cardiovascular disease	0	0
Crohn’s disease	0	0
Chronic fatigue syndrome—Myalgic encephalomyelitis	10/190 (5.2%)	1/77 (1.3%)
Depression requiring medication or therapy	68/190 (35.8%)	29/77 (38%)
Diabetes requiring diet control	3/190 (1.6%)	0
Diabetes requiring insulin or tablets	1/190 (0.5%)	0
Eczema	32/190 (16.8%)	16/77 (21%)
Uterine fibroids	28/190 (9.5%)	7/77 (9.1%)
Fibromyalgia	4/190 (2%)	1/77 (1.3%)
Glandular fever	17/190 (8.9%)	5/77 (6.5%)
Graves’s disease	0	0
Hashimoto’s disease	0	0
High blood pressure	9/141 (6%)	4/64 (6.2%)
Irritable bowel syndrome	43/190 (23%)	17/77 (22%)
Interstitial Cystitis	12/190 (62.5%)	7/77 (9%)
Migraine	51/190 (27%)	22/77 (28.6%)
Mitral valve prolapse	2/190 (1%)	0
Multiple sclerosis	2/190 (1%)	0
Ovarian cysts	93/190 (49%)	27/77 (35.1%)
Pelvic inflammatory disease	13/190 (6.84%)	6/77 (7.8%)
Polycystic ovarian syndrome	32/190 (16.8%)	17 (22.1%)
Rheumatoid arthritis	0	0
Sjogren’s syndrome	0	1/77 (1.3%)
Systemic lupus erythematosus	0	0
Thyroid disease	3/190 (1.6%)	0
Ulcerative colitis	1/190 (0.5%)	2/77 (2.6%)
BMI ≤ 22.5 (underweight/lower-normal)	56/201 (27.9%)	23/75 (30.7%)
BMI > 25 ≤ 30 (overweight)	57/201 (28.3%)	25/75 (33.3%)
BMI > 30 (obese)	40/201 (19.9%)	16/75 (21.3%)

## Data Availability

Data will be available upon request to authors.
